# Supplementary figures and images for: Insight into the Phylogeny and Binding Ability of WRKY Transcription Factors
Source: Int J Mol Sci. 2022 Mar 7;23(5):2895. doi: 10.3390/ijms23052895 (PMC8911475; doi:10.3390/ijms23052895)

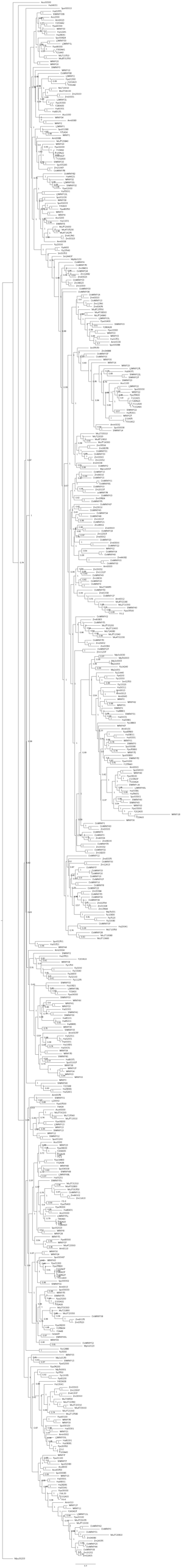

Supplement: Supplementary file 1 [file ijms-23-02895-s001.zip › Figure S1.png]
